# Spastic Ataxia Composite (SPAXCOM): A Scale to Evaluate the Progression of Subjects with Spasticity and Ataxia

**DOI:** 10.1002/mds.70006

**Published:** 2025-08-20

**Authors:** Cécile Di Folco, Charlotte Dubec‐Fleury, Andreas Traschütz, Christoph Kessler, Selina Reich, Cynthia Gagnon, Isabelle Lessard, Xavier Rodrigue, Sirio Cocozza, Sara Satolli, Filippo M. Santorelli, Alexandra Durr, Anna Heinzmann, Bart P. van de Warrenburg, Ilse H.J. Willemse, A. Nazli Başak, Atay Vural, Bernard Brais, Stephan Klebe, Rita Horvath, PROSPAX Consortium, May Yung Tiet, Heather Biggs, Emma Harrison, Dagmar Timmann, Nicole Jeschonneck, Paulina Cunha, Hortense Hurmic, Lucie Pierron, Giulia Coarelli, Claire Ewenczyk, Ivana Ricca, Melissa Barghigiani, Nazan Akkaya, Özgür Öztop Çakmak, Rebecca Schüle, Matthis Synofzik, Sophie Tezenas du Montcel

**Affiliations:** ^1^ Sorbonne Université, Institut du Cerveau‐Paris Brain Institute‐ICM, CNRS, Inria, Inserm, AP‐HP, Groupe Hospitalier Sorbonne Université Paris France; ^2^ Center for Neurology and Hertie Institute for Clinical Brain Research University of Tübingen Tübingen Germany; ^3^ Groupe de Recherche Interdisciplinaire Sur Les Maladies Neuromusculaires (GRIMN), Centre Intégré Universitaire de Santé et de Services Sociaux du Saguenay‐Lac‐St‐Jean Saguenay Quebec Canada; ^4^ Faculté de médecine et des sciences de la santé Université de Sherbrooke Sherbrooke Quebec Canada; ^5^ Centre ÉCOBES‐Recherche et Transfert Cégep de Jonquière Saguenay Quebec Canada; ^6^ Centre Intégré Universitaire de Santé et de Services Sociaux de la Capitale Nationale Québec City Quebec Canada; ^7^ IRCCS Fondazione Stella Maris Pisa Italy; ^8^ Department of Neurology Radboud University Medical Center Nijmegen The Netherlands; ^9^ Koç University, School of Medicine, Research Center for Translational Medicine Istanbul Turkey; ^10^ Department of Neurology Koç University, School of Medicine Istanbul Turkey; ^11^ Montreal Neurological Institute and Hospital, McGill University Montreal Quebec Canada; ^12^ Department of Neurology University Hospital Essen Essen Germany; ^13^ Department of Neurology, Knappschaftkrankenhaus Recklinghausen Recklinghausen Germany; ^14^ Department of Clinical Neurosciences University of Cambridge Cambridge UK; ^15^ Division of Neurodegenerative Diseases and Movement Disorders, Department of Neurology Heidelberg University Hospital and Faculty of Medicine Heidelberg Germany

**Keywords:** ataxia, disease progression, spastic ataxia, spastic ataxia Charlevoix‐Saguenay type, SPG7

## Abstract

**Background:**

Current clinical scales that track disease progression are more tailored to spasticity or ataxia, with limited sensitivity to change.

**Objectives:**

The aim was to develop a sensitive and valid scale specifically geared towards optimized sensitivity to change and adapted to patients presenting with both spasticity and ataxia.

**Methods:**

Longitudinal data from 127 spastic paraplegia type 7 (SPG7) and 112 autosomal recessive spastic ataxia Charlevoix‐Saguenay (ARSACS) patients were collected within the multicenter PROSPAX study. Sensitivity to change over 2 years of 30 items from the Scale for the Rating and Assessment of Ataxias (SARA), Spastic Paraplegia Rating Scale (SPRS), and the Activities of Daily Living subscale of the Friedreich's Ataxia Rating Scale (FARS‐ADL) was evaluated. Items that demonstrated the highest sensitivity to change were used to build the Spastic Ataxia Composite scale (SPAXCOM).

**Results:**

With seven items, the SPAXCOM showed an effect size of 0.71, higher than reference scales (SARA: 0.43, SPRS: 0.42, FARS‐ADL: 0.27). The SPAXCOM had a similar sensitivity to change for both genotypes and was more sensitive in participants with a SARA lower than 10 and within the intermediate disease stage (FARS‐Disease Staging: 2–3.5). The SPAXCOM showed a high correlation with disease duration (*r* = 0.67 [0.60; 0.72]) and disease stage (*r* = 0.86 [0.83; 0.89]). Perception of improvement, stagnation, and worsening were associated with a mean yearly SPAXCOM change of 0.44 (−0.14; 1.01), 0.61 (0.19; 1.03), and 1.22 (0.96; 1.49), respectively.

**Conclusion:**

The SPAXCOM is more sensitive to change and homogeneous across genotypes than the reference scales, allowing a reduction of the required sample size in future clinical trials. © 2025 The Author(s). *Movement Disorders* published by Wiley Periodicals LLC on behalf of International Parkinson and Movement Disorder Society.

Spastic ataxia (SPAX) is a clinical phenotype defined by the coexistence of cerebellar ataxia and spastic paraplegia comprising a heterogeneous group of >100 predominantly hereditary conditions.^1^, ^2^ Among these, autosomal recessive spastic ataxia of Charlevoix‐Saguenay (ARSACS) and spastic paraplegia type 7 (SPG7) are amongst the most common genetic forms of SPAXs worldwide.^2^, ^3^ These diseases are multisystemic, neurodegenerative, and characterized by progressive cerebellar ataxia, spasticity.^4^

With continuous progress in the understanding of the molecular pathogenesis of these and related disorders, novel targeted disease‐modifying therapies are on the horizon and will need to be evaluated in clinical trials. Conducting such trials in these diseases is, however, challenging due to their rarity, heterogeneity, and slow progression. Sensitive outcome measures that capture change in relatively short frames are thus crucial to successful drug development.

Several scales have been published to measure ataxia^5^ and spasticity.^6^ Among those, spasticity and ataxia are often measured by two different scales, the Spastic Paraplegia Rating Scale (SPRS)^7^ and the Scale for the Assessment and Rating of Ataxia (SARA).^8^ As a result, none of the scales are designed for the conditions in which both symptoms are present in combination. SARA showed a low sensitivity to change in SPG7,^3^, ^9^, ^10^ other hereditary spastic paraplegias,^11^ and ARSACS.^3^, ^9^, ^12^, ^13^ SPRS likewise showed a slow sensitivity to change in hereditary spastic paraplegia type 3A, 4, and 5, the latter with prominent ataxia.^14^, ^15^, ^16^ However, their sensitivity to change has not been systematically assessed and compared against each other in genotypes such as SPG7 and ARSACS. The last scale used to monitor disease progression is Friedreich's Ataxia Rating Scale (FARS), comprising, among others, a functional disability staging (FARS‐DS) and a scale evaluating activities of daily living (FARS‐ADL).^17^ The latter was shown to have a similar sensitivity to change to SARA in patients of various genotypes with ataxia.^18^

To address these limitations, our study aimed to build a composite score (the Spastic Ataxia Composite scale [SPAXCOM]) capturing the progression of individuals with spastic ataxia that is sufficiently sensitive to change to serve as an endpoint, using items from the three reference scales assessed in a large longitudinal cohort.

## 1 Patients and Methods

This study was conducted using data acquired within the PROSPAX project (ClinicalTrials.gov No. NCT04297891) approved by the local ethics committee of each center following the ethical standards of the institutional research committee and with the 1964 Helsinki Declaration and its later amendments. Written informed consent was obtained from each patient before enrollment.

### 1.1 Survey Design and Participants

Participants were enrolled in the PROSPAX study (an integrated, multimodal, progression chart in spastic ataxias)—a 2‐year prospective, international, longitudinal, multicenter, natural progression study in spastic ataxias. Participants were included from nine sites in seven countries (Germany, Italy, Canada, Turkey, the Netherlands, France, and the UK). Inclusion criteria were confirmed pathogenic biallelic mutations in SACS or SPG7, clinically manifest disease, age above 10 years, and informed consent (by the patient or by both legal representatives for minors). Participants already enrolled in an interventional study at baseline or with a history of the presence of another neurological disorder were excluded. Healthy controls with no neurological or psychiatric disease history, no family history of neurodegenerative disease, and no neurological signs upon clinical examination were also recruited. Participants underwent clinical evaluation at baseline, 1 year, and 2 years after baseline.

### 1.2 Outcomes

Of the multiple variables measured during the study, this analysis focused on SPRS,^7^ SARA,^8^ and FARS‐ADL.^17^ Items included in each scale are detailed in Table S1. The disease stage was evaluated using the Functional Staging for Ataxia (FARS‐Disease Staging, FARS‐DS).^17^ Based on clinical expertise, the following classification was used: Mild stage for scores from 0 (Normal) to 1.5, Intermediate stage for scores from 2 to 3.5, and Advanced stage for those from 4 to 6 (confined to wheelchair or bed). Age at onset refers to age at motor symptoms onset. Disease duration was computed as the difference between age at baseline visit and age at onset.

### 1.3 Statistical Analysis

**FIG. 1:**
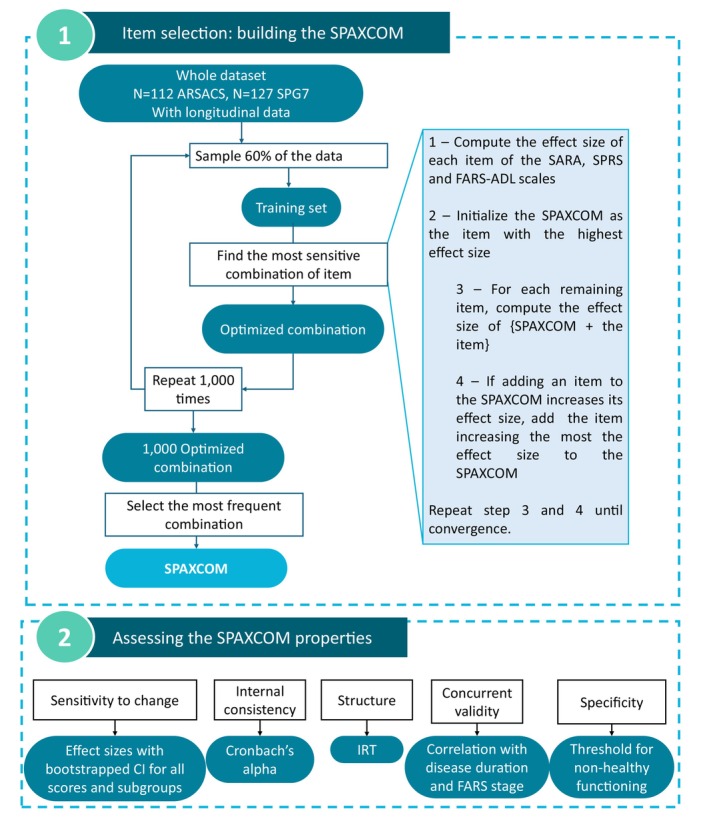
Graphical summary of the methodology to build and assess the Spastic Ataxia Composite scale (SPAXCOM). ARSACS, autosomal recessive spastic ataxia of Charlevoix‐Saguenay; SARA, Scale for the Rating and Assessment of Ataxias; SPRS, Spastic Paraplegia Rating Scale; FARS‐ADL, Friedreich's Ataxia Rating Scale‐Activities of Daily Living; CI, confidence interval; IRT, item response theory. [Color figure can be viewed at wileyonlinelibrary.com]

Our methodology was designed to construct a composite score capturing the progression of individuals with spastic ataxia geared towards optimizing sensitivity to change (Fig. 1). In the first step, we built the SPAXCOM as the combination of items that yielded the highest effect size. Then, we assessed the SPAXCOM sensitivity to change in different subgroups and compared it with those of the reference scales. We further assessed a pathological threshold derived from controls values, structure, internal consistency, and concurrent validity of our composite score.

#### 1.3.1 Item Selection

From the 30 items composing SARA, SPRS, and FARS‐ADL, we aimed to build a combination of items that optimized sensitivity to change. From the complete dataset, we generated 1000 resamples with a stratified random sampling on genotype and FARS Stage. Each resample was composed of a “training” set (60% of the resample participants without replacement) and a “test” set (the remaining 40% of the data). The training set was used to select the item combination yielding the maximal effect size, through a stepwise process detailed in the next paragraph. We call this combination of items “optimized combination.” Then, the test set allowed us to estimate the optimized combination performance on unseen data. As 1000 resamples were drawn, we generated as many (potentially redundant) optimized combinations. The most common of these was kept as the SPAXCOM.

To find an optimized combination of items with the best sensitivity to change on a training set we performed the following steps. First, we computed the effect size of each SARA, SPRS, and FARS‐ADL item as the mean annual change in item score divided by its standard deviation. The annual change in item score was estimated on each patient separately, with a linear regression. Then, starting with the item with the largest effect size, items were added to the composite score one at a time if they increased the effect size of the total score, calculated as the sum of the selected items (see Method 1 in Data S1).

The effect sizes reached by all optimized combinations on their training and test set are reported as computed on all patients, and then separately on the two genotypes (ARSACS and SPG7), on SARA score at baseline (below or over 10), and on the two‐binned FARS Stages (Intermediate and Advanced). As there were too few Mild patients, we did not compute their mean effect size. Lastly, we reported the number of times each item was present in an optimized combination over the 1000 resamples.

#### 1.3.2 Assessment of SPAXCOM Properties

##### 1.3.2.1 Sensitivity to Change

To compare the SPAXCOM performance to that of SARA, SPRS, and FARS‐ADL, we computed its mean effect size with bootstrapped 95% confidence intervals (computed on 500 samples of 50% of the data with replacement). We reported the effect sizes computed on all patients, separately for the two genotypes, for the two‐binned FARS Stages, and for patients with a baseline SARA below and over 10. Patient‐perceived change over each year of the study was measured with the Patient Global Impression of Change Scale (PGI‐C), with its seven levels categorized, before performing the statistical analysis, as follows: Improvement (0 = Very much improved, 1 = Much improved, 2 = Minimally improved), Stable (3 = No change), Worsen (4 = Minimally worse, 5 = Much worse, 6 = Very much worse). We reported, for each PGI‐C value, the average variation in SPAXCOM over each subject and year.

##### 1.3.2.2 Threshold for Non‐Healthy Functioning

To determine the SPAXCOM threshold characterizing a non‐healthy state, we reported the 95th percentile of SPAXCOM obtained from healthy controls using all the available time points.

##### 1.3.2.3 Structure

The structure of the SPAXCOM (ie, how the items correlate with each other) was investigated with an item response theory (IRT) model. The optimal number of unobserved dimensions (latent traits) was chosen based on the Bayesian information criterion (BIC). To assess the quality and appropriateness of the model, and as the studied items are polytomous, we used Cai and Monroe's C2 statistic to compute the fit indices.^17^ We report the root mean square error of approximation (RMSEA), the standardized root mean squared error (SRMSR), the Tucker‐Lewis index (TLI), and the comparative fit index (CFI). A RMSEA lower than 0.06 and a SRMSR lower than 0.08 were considered a good fit. For the CFI and TLI, a value superior to 0.95 was considered a good fit.^18^ We also report the total variance explained by the model and the variance explained at the item level (communalities).

##### 1.3.2.4 Internal Consistency

We assessed the internal consistency of the SPAXCOM total score using Cronbach's alpha, and checked that no items, when removed, led to an increase of the Cronbach's alpha.

##### 1.3.2.5 Concurrent Validity

Correlation of SPAXCOM with disease duration and the FARS Stage was tested using Pearson correlation coefficients. We compared these coefficients with those of SARA, SPRS, and FARS‐ADL with these variables.

#### 1.3.3 Participant Description

We reported baseline characteristics of individuals with ARSACS, SPG7, and healthy controls. Data were expressed as mean ± standard deviation or frequency (percent). Statistical tests were performed at the conventional two‐tailed type I error of 0.05. Data were analyzed using R version 4.3.3 (The R Foundation for Statistical Computing, Vienna, Austria, 2024) for structure assessment and Python 3.8 for the remainder of the analysis.

## 2 Results

### 2.1 Participants

The study sample comprised 127 SPG7 participants, 112 ARSACS participants, and 58 healthy controls for whom clinical data were available at baseline and at the 1‐year follow‐up (Fig. S1). For 93% of the SPG7 participants (118/127), 79% of the ARSACS participants (88/112), and 93% of the healthy controls (54/58), 2‐year follow‐up data were also available.

At baseline, SPG7 participants had a mean age of 55.4 ± 10.2 years and a disease duration of 19.0 ± 10.3 years. Most participants (60%) had intermediate disease (Table 1). ARSACS participants had a mean age of 35.3 ± 12.7 years and a disease duration of 29.5 ± 11.6 years. Most ARSACS participants (53%) had advanced disease. Healthy controls were on average aged 46.6 ± 14.7 years. All controls had a SARA score below 5, SPRS below 6, and FARS‐ADL below 4 points.

**TABLE 1:** Characteristics of participants at baseline

Characteristic	SPG7 (N = 127)	ARSACS (N = 112)	HC (N = 58)
Sex (female)	48 (37.8)	52 (46.4)	32 (55.2)
Age at baseline (years)	55.4 ± 10.2	35.3 ± 12.7	46.6 ± 14.7
Age at onset (years)	36.4 ± 12.6	6.3 ± 9.0	
Disease duration (years)	19.0 ± 10.3	29.5 ± 11.6	
FARS Stage			
Advanced	40 (31.5)	59 (52.7)	
Intermediate	76 (59.8)	39 (34.8)	
Mild	11 (8.7)	14 (12.5)	58 (100.0)
SARA total (/40)	11.71 ± 5.52	17.93 ± 7.54	0.47 ± 0.81
SPRS total (/52)	18.29 ± 8.20	22.27 ± 10.79	0.93 ± 1.36
FARS‐ADL (/36)	11.07 ± 5.25	13.16 ± 6.63	0.17 ± 0.56
SPAXCOM (/32)	10.24 ± 5.29	14.32 ± 7.13	0.31 ± 0.78

Disease duration: age at baseline–age at symptom onset. Mild disease stage: FARS Stage scores 0 to 1.5, Intermediate: scores 2 to 3.5, Advanced: scores 4 to 6. Data are expressed as mean ± standard deviation or frequency (percent).

Abbreviations: ARSACS, autosomal recessive spastic ataxia of Charlevoix‐Saguenay; FARS, Friedreich's Ataxia Rating Scale; FARS‐ADL, Friedreich's Ataxia Rating Scale‐Activities of Daily Living; HC, healthy controls; SARA, Scale for the Rating and Assessment of Ataxias; SPRS, Spastic Paraplegia Rating Scale; SPAXCOM, Spastic Ataxia Composite scale; SPG7, spastic paraplegia type 7.

### 2.2 SPAXCOM

#### 2.2.1 Item Selection

**FIG. 2:**
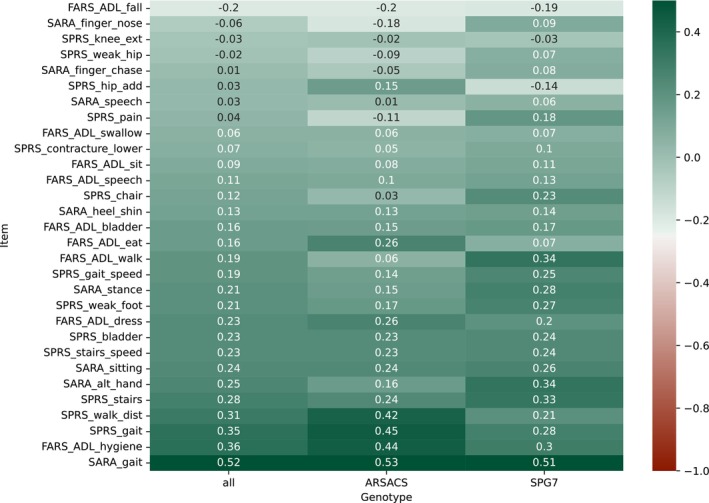
Effect sizes of the 30 items for the Scale for the Rating and Assessment of Ataxias (SARA), Spastic Paraplegia Rating Scale (SPRS), and Friedreich's Ataxia Rating Scale‐Activities of Daily Living (FARS‐ADL) scales. Effect sizes are reported for spastic paraplegia type 7 (SPG7) + autosomal recessive spastic ataxia of Charlevoix‐Saguenay (ARSACS) participants (all) and for each genotype separately. Items are sorted by their effect size on all participants. The effect size is the mean standardized annual change in score, calculated using linear regression for each patient separately. [Color figure can be viewed at wileyonlinelibrary.com]

All longitudinal data from 127 SPG7 participants and 112 ARSACS participants were used for item selection. Effect sizes of the items ranged from −0.2 to 0.51 for ARSACS and SPG7 participants combined, with 12 items displaying an effect size superior to 0.2 (Fig. 2).

Over the 1000 data resamples on which the stepwise item selection was performed, 161 different optimized combinations of items were obtained, of the 2^30 theoretically possible combinations of 30 items. These combinations reached an average effect size of 0.77 (2.5th and 97.5th percentiles: [0.66; 0.90]) on training data and (0.55 [0.36; 0.74]) on test data (Fig. S2).

The most frequent optimized combination occurred 216 times (including 205 times in association with other items, and 11 times alone) (Fig. S3). It was composed of seven items: three items from SARA (gait, sitting, and fast alternating hand movements), three items from SPRS (walking distance without pause, climbing stairs, and weakness of foot dorsiflexion), and one from FARS‐ADL (personal hygiene). These items were also among the most frequently selected items over the 1000 iterations: four of them (SARA gait, SARA fast alternating hand movements, SPRS walking distance without pause, FARS‐ADL personal hygiene) were selected more than 90% of the times, two (SPRS climbing stairs and SPRS weakness of foot dorsiflexion) were selected more than 65% of the time, and one (SARA sitting) was selected 49% of the time (Fig. S4). The SPAXCOM score was obtained by summing these seven items, with a range from 0 (no disability) to 32 (maximum disability) (Data S1).

#### 2.2.2 Sensitivity to Change

**FIG. 3:**
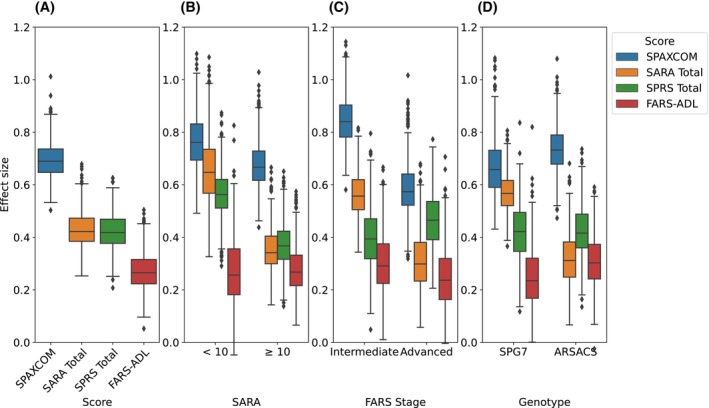
Effect sizes for the Spastic Ataxia Composite (SPAXCOM), Scale for the Rating and Assessment of Ataxias (SARA), Spastic Paraplegia Rating Scale (SPRS), and Friedreich's Ataxia Rating Scale‐Activities of Daily Living (FARS‐ADL) total scores, and their bootstrapped confidence intervals. (A) On all data. (B) Assessed separately according to baseline SARA scores. (C) Assessed separately on patients’ FARS Stage. The boxplot corresponding to patients with Mild stage is not shown, due to insufficient data (N = 25). Whiskers correspond to 1.5 times the interquartile range. (D) Assessed separately on both genotypes. [Color figure can be viewed at wileyonlinelibrary.com]

The SPAXCOM score achieved a mean effect size of 0.71 (95% bootstrapped CI: 0.58; 0.85) for ARSACS and SPG7 participants combined (Fig. 3A, Table S2). This corresponds to ARSACS participants increasing on average by 1.10 (0.82; 1.34) points of SPAXCOM per year, and SPG7 participants increasing on average by 1.00 (0.73; 1.27) points of SPAXCOM per year. By comparison, SARA reached a mean effect size of 0.43 (0.31; 0.59), SPRS of 0.42 (0.3; 0.56), and FARS‐ADL of 0.27 (0.14; 0.43). The mean (95%) change in SPAXCOM score was 0.44 (−0.14; 1.01) for subjects reporting an improvement over the last year, 0.61 (0.19; 1.03) for subjects reporting being stable, and 1.22 (0.96; 1.49) for those reporting a worsening condition. Among the 76 controls with a stable PGI‐C, the mean SPAXCOM evolution was −0.01 (−0.1; 0.08) (95% CI) (Table S3).

#### 2.2.3 Subgroup Analysis

On patients with a SARA score below 10 at baseline, the SPAXCOM had an average effect size of 0.77 (0.59; 0.99), similar to that of patients with a baseline SARA score over 10 (0.68 [0.53; 0.89]) (Fig. 3B). When we used the FARS Stage to separate the patients, the average effect size was 0.86 (0.7; 1.05) in participants at the Intermediate stage, and 0.60 (0.43; 0.85) in participants at the Advanced stage (Fig. 3C). The effect size of the SPAXCOM was similar in both genotypes: 0.75 (0.58; 0.93) and 0.69 (0.51; 0.95) in ARSACS and SPG7 participants, respectively (Fig. 3D). SPRS and FARS‐ADL also had similar effect sizes for both genotypes and disease stages (Table S2). SARA, however, showed a higher sensitivity to change in SPG7 than in ARSACS, and in Intermediate‐stage participants than in Advanced‐stage ones. Compared with the SPAXCOM, all scores had a lower sensitivity to change in all subgroups, except SARA, which reached a similar effect size to that of the SPAXCOM on SPG7 patients and patients with milder ataxia (SARA below 10). Finally, the SPAXCOM of healthy controls remained stable, with a mean effect size of −0.12 (−0.29; 0.19).

#### 2.2.4 Threshold for Non‐Healthy Functioning

**FIG. 4:**
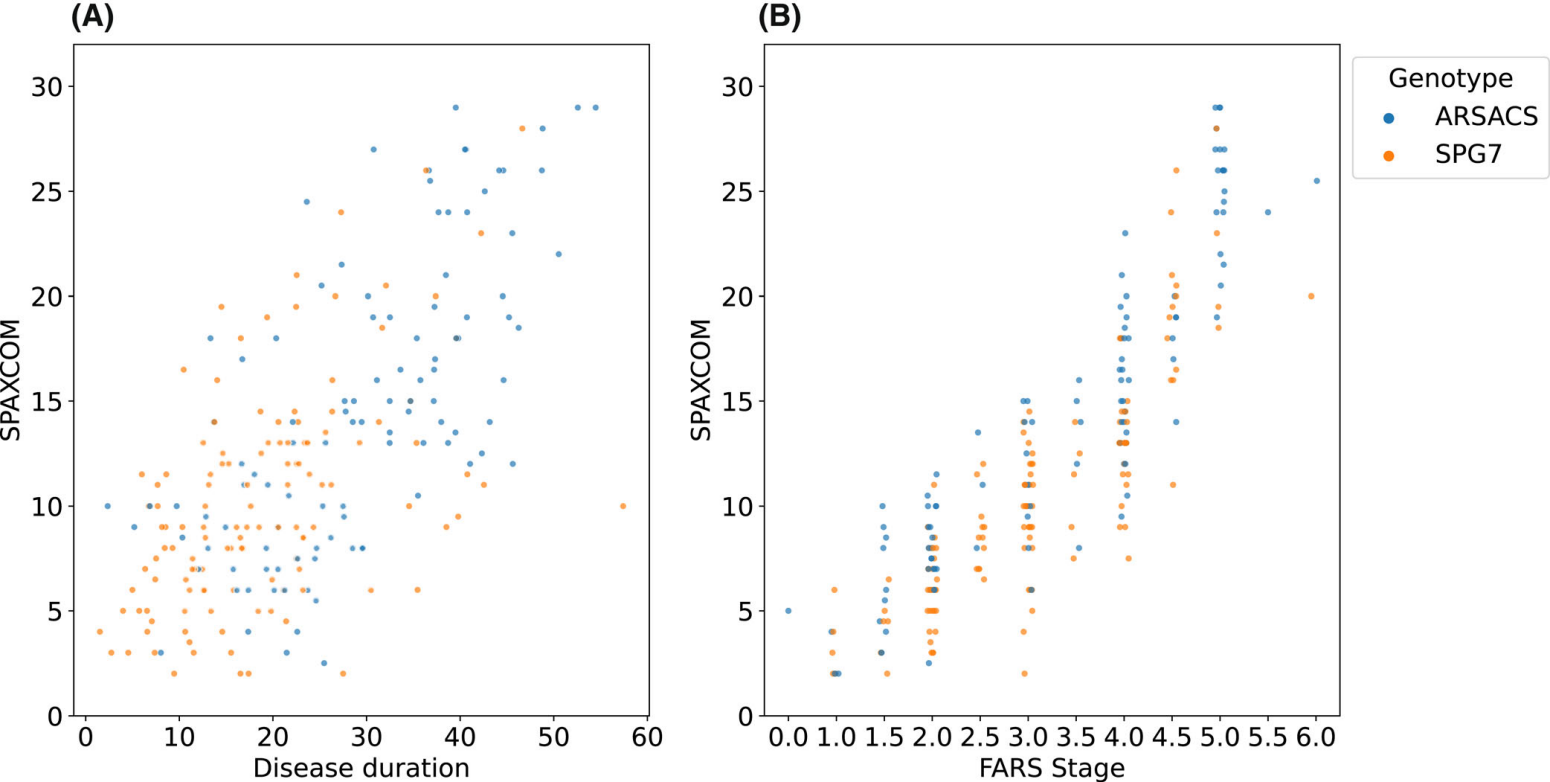
Correlation between the Spastic Ataxia Composite scale (SPAXCOM), disease duration, and disease stage. (A) Scatterplot displaying the SPAXCOM score depending on disease duration, computed as the difference between age at baseline visit and age at symptoms onset (N = 232). (B) Scatterplot displaying the SPAXCOM score depending on Friedreich's Ataxia Rating Scale (FARS) Stage (N = 239). [Color figure can be viewed at wileyonlinelibrary.com]

For healthy controls, the SPAXCOM values ranged from 0 to 5, with a mean value of 0.31 ± 0.78; 95% of them had a SPAXCOM value below 1. A threshold value of 1 can thus be chosen to characterize a healthy state. At baseline, the average SPAXCOM total score was 10.24 ± 5.29 in SPG7 participants and 14.32 ± 7.13 in ARSACS participants, none being below the score value of 2 points (Fig. 4).

#### 2.2.5 Structure

Given the small number of items of the SPAXCOM, unidimensional and two‐dimensional IRT models were tested. The unidimensional model displaying a lower BIC was retained (1‐dimension BIC: 3778, 2‐dimension BIC: 3796). It explained 65% of the score variance with fair fit indices (RMSEA = 0.100 [0.068, 0.132], CFI = 0.979, TLI = 0.969, SRMSR = 0.058; Table S4).

#### 2.2.6 Internal Consistency

The SPAXCOM showed good internal consistency, with a Cronbach's alpha of 0.878 (0.844; 0.906). One item increased the Cronbach's alpha when removed (SARA alternating hand), but not out of the confidence interval (Table S4). The seven items were correlated, with correlations ranging from 0.3 to 0.8 (Fig. S5).

#### 2.2.7 Concurrent Validity

The SPAXCOM score was highly correlated with disease duration (*r* = 0.67, [0.60; 0.72], *P* < 0.001, Fig 4A), as was the SARA (*r* = 0.71 [0.65; 0.76], *P* < 0.001) and, to a lesser extent, the SPRS (*r* = 0.55 [0.47; 0.62], *P* < 0.001) and FARS‐ADL scores (*r* = 0.58 [0.5; 0.65], *P* < 0.001). Of all the scores, the SPAXCOM had the highest correlation with the FARS‐DS (*r* = 0.86 [0.83; 0.89], *P* < 0.001, Fig 4B). A high correlation with FARS‐DS was also found for SARA (*r* = 0.79 [0.75; 0.83], *P* < 0.001), SPRS (*r* = 0.79 [0.74; 0.82], *P* < 0.001), and FARS‐ADL (*r* = 0.76 [0.71; 0.8], *P* < 0.001).

## 3 Discussion

This study aimed to develop a composite score for spastic ataxia, specifically geared towards optimized sensitivity to change and adapted to patients presenting both spasticity and ataxia. The novel SPAXCOM allows capture with a single scale of the progression of participants with spastic ataxia with higher sensitivity to change than the tested reference scales, rendering it the currently most promising clinician‐reported outcome (ClinRO) in clinical trials for spastic ataxias.

To generate a common, change‐optimized scale for genotypes presenting with spastic ataxia, we combined the most sensitive items of the FARS‐ADL, SPRS, and SARA scores. Our primary rationale for selecting items based on sensitivity to change was to maximize the responsiveness and efficiency of the outcome measure in clinical trial settings. This intended context of use justifies, even necessitates, a more focused, composite approach as it enhances the ability to detect treatment effects by concentrating on the most dynamic and clinically relevant features. Similar approaches have been applied before, for example, just recently with the development of the SCACOMS, a composite scale for spinocerebellar ataxias derived from items from the Clinical Global Impression‐Global Improvement Scale (CGI‐I), the Friedreich Ataxia Rating Scale (FARS)‐Functional Stage, and the Modified Functional Scale for the Assessment and Rating of Ataxia (f‐SARA) (https://doi.org/10.1007/s12311-024-01697-8).^19^ The SPAXCOM was developed following similar principles, combining the most sensitive and representative items to better capture disease progression. The resulting scale (SPAXCOM) comprised three items from SARA (gait, sitting, and fast alternating hand movements), three items from SPRS (walking distance without pause, climbing stairs, and weakness of foot dorsiflexion), and one from FARS‐ADL (personal hygiene). Three items of the seven directly assessed mobility (SPRS stairs, walking distance, SARA gait), one from SARA assessed the trunk control (SARA sitting), one from SPRS assessed lower limb weakness, one from SARA assessed kinetic function of the upper limb, and one from FARS‐ADL evaluated personal hygiene activities. Interestingly, the latter has been shown to be gait‐ and stance‐related.^16^ These items strongly correlated, as reflected by the good internal consistency and the fair unidimensional structure. Only the fast‐alternating hand movement item from SARA did not strongly correlate with the others. The low correlation for this item might be explained by the fact that it assesses an effector domain (upper limb function) at least partly distinct from the other effector domains. As this item captures an important domain of patient daily living, and this item showed a strong sensitivity to change, it was retained in the final SPAXCOM. The two SPRS items that measure spasticity (items 7 and 8) were not included in the SPAXCOM. This is in line with a recent review that highlighted the difficulty of measuring spasticity with these two items.^6^

With only these seven items, the composite score showed a higher sensitivity to change than the widely used current reference scales for ataxia (SARA) and spasticity (SPRS). This is of importance as also the SARA score is partly influenced by non‐ataxia features (eg, SARA item gait by gait spasticity), and the SPRS by non‐spasticity features (eg, the SPRS gait items by gait ataxia), respectively. However, this unspecific influence by neurological system deficits for which the respective scale has not been primarily designed does not seem to help a lot with sensitivity to change in diseases with combined ataxia and spasticity.

Importantly, the SPAXCOM was as sensitive in participants with ARSACS as in those with SPG7. In contrast, SARA was more sensitive to change in participants with SPG7 than ARSACS. Interestingly, many items in the three scales had different sensitivity to change for the two genotypes. The SPAXCOM is composed of two items with similar effect sizes in both genotypes (SARA gait and sitting), two items with higher sensitivity in ARSACS than in SPG7 (SPRS walking distance, FARS‐ADL personal hygiene), and three items more sensitive in SPG7 than in ARSACS (SARA alternating hand, SPRS stairs and weak foot). This contributes to the usefulness of SPAXCOM in both diseases. FARS‐ADL hygiene and walk were found to be the most sensitive to change among all the items of the FARS‐ADL in patients with spinocerebellar ataxias.^16^ As for SARA items, gait was shown to be the most sensitive one.^16^, ^20^, ^21^ According to studies in autosomal dominant^20^, ^21^ and autosomal recessive^22^ ataxias, gait and stance assessments are sensitive at the beginning of the disease, while sitting is impaired at later stages, and alternating hand movements show changes in the middle of the disease course. The SPAXCOM comprises items informing disease progression at different stages. Overall, the SPAXCOM showed a higher sensitivity in participants with a SARA score below 10 at baseline and those at intermediate disease stages than in those at advanced disease stages. This was also the case for SARA, but not for SPRS or FARS‐ADL. This is consistent with recent work showing that in polyQ spinocerebellar ataxias, SARA was less sensitive to change for patients at an advanced disease stage compared with those at mild and intermediate stages.^20^ Thus, the SPAXCOM could be a valuable scale in clinical trials targeting patients in the mild or intermediate stage of the disease, which is of greater interest for trials than the advanced stage, as neurodegeneration is less advanced and stabilization or even reversibility of the disease may be achievable.

To further demonstrate the validity of the composite scale, we showed the highest correlation with FARS disease stage and with disease duration. The relationship with disease duration of the SPRS was like those found with patients with hereditary spastic ataxias.^14^ For patients with Friedreich ataxia, the correlation between SARA and disease duration was like the one in our study.^23^

To our knowledge, this study is the first that has evaluated the sensitivity to change of three reference scales in a large sample of ARSACS and SPG7 participants. The 2‐year follow‐up and the three assessment time points also constitute strengths to accurately estimate patients' progression and are seldom available for such rare diseases. Using anchor‐based methods, we have shown that the SPAXCOM was responsive for patients with both genotypes. The effect sizes from the current study need to be confirmed with longer follow‐up if used in longer clinical trials.

This study nonetheless has limitations. To build the composite score, a compromise had to be made between structure (ie, how items correlate together) and sensitivity to change. As sensitivity to change is typically the limiting factor of outcome measures for slowly progressive diseases, we prioritized it over structure. With only seven items, the SPAXCOM is a new tool to monitor disease progression in individuals with spastic ataxia with a higher sensitivity to change than the current reference scales, allowing a reduction of the required sample size in future clinical trials. It shows homogeneous properties across genotypes. SPAXCOM is also highly correlated with disease stage and disease duration. A replication cohort is needed to validate this scale and allow its use as an endpoint in progression studies and treatment trials. The SPAXCOM scale may also be a promising outcome measure for other spastic ataxias, such as spinocerebellar ataxia.

## Author Roles

(1) Research Project: A. Conception, B. Organization, C. Execution; (2) Statistical analysis: A. Design, B. Execution, C. Review and Critique; (3) Manuscript: A. Writing of the First Draft, B. Review and Critique.

C.D.F.: 2B, 3A.

C.D.‐.F.: 2B, 3B.

A.T.: 1C, 3B.

C.K.: 1C, 3B.

S.R.: 2B, 3B.

C.G.: 1C, 3B.

I.L.: 1C, 3B.

X.R.: 1C, 3B.

S.C.: 1C, 3B.

S.S.: 1C, 3B.

F.M.S.: 1C, 3B.

A.D.: 1C, 3B.

A.H.: 1C, 3B.

B.P.v.d.W.: 1C, 3B.

I.H.J.W.: 1C, 3B.

A.N.B.: 1C, 3B.

A.V.: 1C, 3B.

B.B.: 1C, 3B.

S.K.: 1C, 3B.

R.H.: 1C, 3B.

R.S.: 1A, 2A, 2C, 3B.

M.S.: 1A, 2A, 2C, 3B.

S.T.d.M.: 1A, 2A, 2C, 3A.

## Financial Disclosures of All Authors (for the Previous 12 Months)

C.D.F., C.D.‐.F., A.T., C.K., S.R., I.L., X.R., S.S., F.M.S., A.H., I.H.J.W., A.N.B., A.V., B.B., S.K., R.H., and R.S. declare no financial disclosure. C.G. has received funding from the Fondation de l'Ataxie de Charlevoix‐Saguenay, Canadian Institutes of Health Research and Muscular Dystrophy Canada for projects related to the present work. C.G. has served on advisory boards and received consultancy fees from Dyne Therapeutics unrelated to the present work; and received research grants from Canadian Institutes of Health Research, European Joint Program on Rare Diseases, Muscular Dystrophy Canada, Association Française Contre les Myopathies, and Vertex Pharmaceuticals. S.C. consulted for TTLC SRL, has served on the scientific advisory board of Amicus Therapeutics, and has received research grants from FISM and Telethon. A.D. declares that her institution (Paris Brain Institute) receives consulting fees on her behalf from Huntix, UCB, Biogen, and PTC Therapeutics as well as research grants from the National Institutes of Health (NIH), Agency for Research (ANR), National Hospital Clinical Research Program (PHRC), and Foundation of Recherche Médicale (FRM), and partly holds a Patent B 06291873.5 on “Anaplerotic therapy of Huntington's disease and other polyglutamine diseases”. B.P.v.d.W. has served on advisory boards and/or received consultancy fees from Biogen, Biohaven Pharmaceuticals, Servier, and Vico Therapeutics, all unrelated to the present work. M.S. has received consultancy honoraria from Ionis, UCB, Prevail, Orphazyme, Servier, Reata, GenOrph, AviadoBio, Biohaven, Solaxa, Biogen, Zevra, and Lilly, all unrelated to the present work. S.T.d.M. has received consultancy honoraria from Vico Therapeutics unrelated to the present work.

## Supporting information


**Data S1.** Supporting Information.

## Data Availability

The data that support the findings of this study are available on request from the corresponding author. The data are not publicly available due to privacy or ethical restrictions.

## Appendix A

May Yung Tiet^1^, Heather Biggs^1^, Emma Harrison^1^, Dagmar Timmann^2^, Nicole Jeschonneck^2^, Paulina Cunha^3^, Hortense Hurmic^3^, Lucie Pierron^3^, Giulia Coarelli^3^, Claire Ewenczyk^3^, Ivana Ricca^4^, Melissa Barghigiani^4^, Nazan Akkaya^5^, Özgür Öztop Çakmak^6^.

^1^Department of Clinical Neurosciences, University of Cambridge, Cambridge, UK.

^2^Department of Neurology, University Hospital Essen, Essen, Germany.

^3^Sorbonne Université, Institut du Cerveau‐Paris Brain Institute‐ICM, CNRS, Inria, Inserm, AP‐HP, Groupe Hospitalier Sorbonne Université, Paris, France.

^4^IRCCS Fondazione Stella Maris, Pisa, Italy.

^5^ Koç University Translational Medicine Research Center (KUTTAM), Koc University, Istanbul, Turkey.

^6^Department of Neurology, Koç University, Istanbul, Turkey.

## References

[1] Synofzik M , Schüle R . Overcoming the divide between ataxias and spastic paraplegias: shared phenotypes, genes, and pathways. Mov Disord 2017;32(3):332–345. 28195350 10.1002/mds.26944 PMC6287914

[2] Pedroso JL , Vale TC , França Junior MC , Kauffman MA , Teive H , Barsottini OGP , Munhoz RP . A diagnostic approach to spastic ataxia syndromes. Cerebellum 2022;21(6):1073–1084. 34782953 10.1007/s12311-021-01345-5

[3] Traschütz A , Adarmes‐Gomez AD , Anheim M , et al. Autosomal recessive cerebellar ataxias in Europe: frequency, onset, and severity in 677 patients. Mov Disord 2023;38(6):1109–1112. 37027459 10.1002/mds.29397

[4] Lallemant‐Dudek P , Darios F , Durr A . Recent advances in understanding hereditary spastic paraplegias and emerging therapies. Fac Rev 2021;10:27. 33817696 10.12703/r/10-27 PMC8009193

[5] Perez‐Lloret S , Warrenburg B , Rossi M , et al. Assessment of ataxia rating scales and cerebellar functional tests: critique and recommendations. Mov Disord 2021;36(2):283–297. 33022077 10.1002/mds.28313

[6] Gal O , Baude M , Deltombe T , et al. Clinical outcome assessments for spasticity: review, critique, and recommendations. Mov Disord 2025;40(1):22–43. 39629752 10.1002/mds.30062 PMC11752990

[7] Schüle R , Holland‐Letz T , Klimpe S , et al. The spastic paraplegia rating scale (SPRS): a reliable and valid measure of disease severity. Neurology 2006;67(3):430–434. 16894103 10.1212/01.wnl.0000228242.53336.90

[8] Schmitz‐Hübsch T , du Montcel ST , Baliko L , et al. Scale for the assessment and rating of ataxia: development of a new clinical scale. Neurology 2006;66(11):1717–1720. 16769946 10.1212/01.wnl.0000219042.60538.92

[9] Traschütz A , Adarmes‐Gómez AD , Anheim M , et al. Responsiveness of the scale for the assessment and rating of ataxia and natural history in 884 recessive and early onset ataxia patients. Ann Neurol 2023;94(3):470–485. 37243847 10.1002/ana.26712

[10] Coarelli G , Schule R , , van de van de Warrenburg B , et al. Loss of paraplegin drives spasticity rather than ataxia in a cohort of 241 patients with SPG7. Neurology 2019;92:e2679–e2690. 31068484 10.1212/WNL.0000000000007606 PMC6556095

[11] Amprosi M , Indelicato E , Eigentler A , Fritz J , Nachbauer W , Boesch S . Toward the definition of patient‐reported outcome measurements in hereditary spastic paraplegia. Neurol Genet 2023;9(1):e200052. 36636734 10.1212/NXG.0000000000200052 PMC9832334

[12] Bourcier D , Bélanger M , Côté I , et al. Documenting the psychometric properties of the scale for the assessment and rating of ataxia to advance trial readiness of autosomal recessive spastic ataxia of Charlevoix‐Saguenay. J Neurol Sci 2020;417:117050. 32736199 10.1016/j.jns.2020.117050

[13] Hendrickx N , Mentré F , Traschütz A , et al. Prediction of individual disease progression including parameter uncertainty in rare neurodegenerative diseases: the example of autosomal‐recessive spastic ataxia Charlevoix Saguenay (ARSACS). AAPS J 2024;26(3):57. 38689016 10.1208/s12248-024-00925-7

[14] Giordani GM , Diniz F , Fussiger H , et al. Clinical and molecular characterization of a large cohort of childhood onset hereditary spastic paraplegias. Sci Rep 2021;11:22248. 34782662 10.1038/s41598-021-01635-2 PMC8593146

[15] Schöls L , Rattay TW , Martus P , et al. Hereditary spastic paraplegia type 5: natural history, biomarkers and a randomized controlled trial. Brain 2017;140(12):3112–3127. 29126212 10.1093/brain/awx273 PMC5841036

[16] Cubillos Arcila DM , Dariva Machado G , Martins VF , et al. Long‐term progression of clinician‐reported and gait performance outcomes in hereditary spastic paraplegias. Front Neurosci 2023;17:1226479. 10.3389/fnins.2023.1226479 37811319 PMC10556702

[17] Subramony SH , May W , Lynch D , et al. Measuring Friedreich ataxia: interrater reliability of a neurologic rating scale. Neurology 2005;64:1261–1262. 15824358 10.1212/01.WNL.0000156802.15466.79

[18] Traschütz A , Fleszar Z , Hengel H , et al. FARS‐ADL across ataxias: construct validity, sensitivity to change, and minimal important change. Mov Disord 2024;39(6):965–974. 38509638 10.1002/mds.29788

[19] L'Italien G , Popoff E , Rogula B , et al. Development and validation of SCACOMS, a composite scale for assessing disease progression and treatment effects in spinocerebellar ataxia. Cerebellum 2024;23(5):2028–2041. 38710966 10.1007/s12311-024-01697-8 PMC11489241

[20] Petit E , Schmitz‐Hübsch T , Coarelli G , et al. SARA captures disparate progression and responsiveness in spinocerebellar ataxias. J Neurol 2024;271(7):3743–3753. 10.1007/s00415-024-12475-1 38822840 PMC11571887

[21] Moulaire P , Poulet PE , Petit E , Klockgether T , Durr A , Ashisawa T , Tezenas du Montcel S . Temporal dynamics of the scale for the assessment and rating of ataxia in spinocerebellar ataxias. Mov Disord 2022;38(1):35–44. Portico. 10.1002/mds.29255 36273394 PMC9851985

[22] Hamdan A , Hooker AC , Chen X , Traschütz A , Schüle R , Synofzik M , Karlsson MO . Item performance of the scale for the assessment and rating of ataxia in rare and ultra‐rare genetic ataxias. CPT Pharmacometrics Syst Pharmacol 2024;13(8):1327–1340. Portico. 10.1002/psp4.13162 38769902 PMC11330187

[23] Marelli C , Figoni J , Charles P , et al. Annual change in Friedreich's ataxia evaluated by the scale for the assessment and rating of ataxia (SARA) is independent of disease severity. Mov Disord 2011;27(1):135–139. Portico. 10.1002/mds.23879 22076850

