# Supplementary material for: Spastic Ataxia Composite (SPAXCOM): A Scale to Evaluate the Progression of Subjects with Spasticity and Ataxia
Source: Mov Disord. 2025 Aug 20;40(11):2476–85. doi: 10.1002/mds.70006 (PMC12661626; doi:10.1002/mds.70006)
Supplement: Supplementary file 1 — Data S1. Supporting Information. [file MDS-40-2476-s001.docx]

# Appendix

**Supplementary methods**

**eMethod 1: Pseudo-code corresponding to the item selection procedure**

**Supplementary Material**

**eMaterial 1: Scoring sheet**

**Supplementary figures**

**eFigure 1: Flowchart illustrating patients’ selection**

**eFigure 2: Effect size of items combinations during the stepwise procedure.**

**eFigure 3: Number of times a combination of items was selected as maximizing effect size during the stepwise procedure.**

**eFigure 4: Number of times each item is selected in an optimized combination during the stepwise procedure on 1,000 data splits. At each iteration, 60% of the data is drawn. The “optimized combination” of items, i.e., the combination maximizing effect size, is then computed in a stepwise procedure. We report the number of times each item was present in the optimized combinations resulting from N=1,000 iterations.**

**eFigure 5: Correlation at baseline between the seven items of the SPAXCOM**

**Supplementary tables**

**eTable 1: Description and labels of SARA, SPRS and FARS-ADL items.**

**eTable 2: Effect sizes for the SPAXCOM, SARA, SPRS and FARS-ADL total scores, and their bootstrapped confidence intervals.**

**eTable 3: Mean SPAXCOM variation [95% CI] by PGI-C.**

**eTable 4: Internal consistency of the SPAXCOM.**

**eMethod 1: Pseudo-code corresponding to the item selection procedure**

For i = 1 to 1,000:

Candidates = all items in SARA, SPRS and FARS-ADL

Optimized_combination = []

Stored_effect_sizes = []

Best_effect_size = 0

Training set = Sample 60% of participants data (stratified on genotype and stage)

Test set = All dataset - Training test

While (Candidates not empty):

New_effect_sizes = []

For candidate in Candidates:

Compute effect size of {Optimized_combination + candidate}

Append effect size to New_effect_sizes

If max(New_effect_sizes) < Best_effect_size :

Stop

Else:

Best_candidate = score corresponding to max(New_effect_sizes)

Remove Best_candidate from Candidates

Append Best_candidate to Optimized_combination

Store max(New_effect_sizes) in Stored_effect_sizes

**eMaterial 1: SPAXCOM Scoring sheet**

**1) Gait (SARA 1)**

Proband is asked (1) to walk at a safe distance parallel to a wall including a half-turn (turn around to face the opposite direction of gait) and (2) to walk in tandem (heels to toes) without support.

0: Normal, no difficulties in walking, turning and walking tandem (up to one misstep allowed)

1: Slight difficulties, only visible when walking 10 consecutive steps in tandem

2: Clearly abnormal, tandem walking >10 steps not possible

3: Considerable staggering, difficulties in half-turn, but without support

4: Marked staggering, intermittent support of the wall required

5: Severe staggering, permanent support of one stick or light support by one arm required

6: Walking > 10 m only with strong support (two special sticks or stroller or accompanying person)

7: Walking < 10 m only with strong support (two special sticks or stroller or accompanying person)

8: Unable to walk, even supported

**Score: _____/8**

**2) Walking distance without pause (SPRS 1)**

Assessment of the proband's walking distance without pause. Due to history, walking aids are allowed.

0: Normal, unlimited [The proband's walking distance is normal and unlimited.]
1: Abnormal exhaustion [The proband experiences abnormal exhaustion due to spasticity after more than 500 metres.]
2: Less than 500 metres [The proband's walking distance is less than 500 metres.]
3: Less than 10 metres [The proband's walking distance is less than 10 metres.]
4: Unable to walk [The proband is unable to walk.]

**Score: _____/4**

**3) Climbing stairs (SPRS 4)**

Assessment of the proband's ability to climb stairs. 5 steps upstairs - turn - 5 steps downstairs.

0: Normal [The proband is able to climb stairs in a normal manner and needs no support of the banister.]
1: Mild impairment [The proband's ability to climb stairs is mildly impaired, and the proband needs intermittent support of the banister.]
2: Moderate impairment [The proband's ability to climb stairs is moderately impaired, and the proband needs permanent support of the banister.]
3: Severe impairment [The proband's ability to climb stairs is severely impaired, and the proband needs support of another person or additional walking aid to perform task.]
4: Unable [The proband is unable to climb stairs.]

**Score: _____/4**

**4) Sitting (SARA 3)**

Proband is asked to sit on an examination bed without support of feet, eyes open and arms outstretched to the front.

0: Normal, no difficulties sitting >10 sec

1: Slight difficulties, intermittent sway

2: Constant sway, but able to sit > 10 s without support

3: Able to sit for > 10 s only with intermittent support

4: Unable to sit for >10 s without continuous support

**Score: _____/4**

**5) Fast alternating hand movements (SARA 7)**

**Rated separately for each side**

Proband sits comfortably. If necessary, support of feet and trunk is allowed. Proband is asked to perform 10 cycles of repetitive alternation of pro- and supinations of the hand on his/her thigh as fast and as precise as possible. Movement is demonstrated by examiner at a speed of approx. 10 cycles within 7 s. Exact times for movement execution have to be taken.

0: Normal, no irregularities (performs <10s)

1: Slightly irregular (performs <10s)

2: Clearly irregular, single movements difficult to distinguish or relevant interruptions, but performs <10s

3: Very irregular, single movements difficult to distinguish or relevant interruptions, performs >10s

4: Unable to complete 10 cycles

Score: **R**ight **_____/4 L**eft **_____/4**

**Score:** Mean of both sides (R+L)/2 **_____/4**

**6) Weakness - foot dorsiflexion (SPRS 10)**

Assessment of weakness in foot dorsiflexion (Medical Research Council 1976).

0: No weakness [The proband exhibits no weakness in foot dorsiflexion.]
1: Mild weakness (4/5) [The proband exhibits mild weakness (4/5) in foot dorsiflexion.]
2: Moderate weakness (3/5) [The proband exhibits moderate weakness (3/5) in foot dorsiflexion.]
3: Severe weakness (2/5) [The proband exhibits severe weakness (1-2/5) in foot dorsiflexion.]
4: Plegia (0/5) [The proband exhibits plegia (0/5) in foot dorsiflexion.]

**Score: _____/4**

**7) How affected is your/ the patient's ability to perform personal hygiene activities by himself/herself? (FARS ADL 5)**

Ideally, this question should be ascertained from the family/ spouse/caregiver. If both the proband and the family member provide a rating and there is disagreement, please enter the family member's score. Increments of 0.5 may be used if strongly felt that a task falls between 2 scores.

How was this collected? □ Participant/family reported only □ Administered/structured interview

Response provided by: □ Participant □ Family/Spouse/Caregiver □ Participant and Family

0: Normal

1: Somewhat slow, but no help needed

2: Very slow hygienic care or has need for devices such as special grab bars, tub bench shower chair, etc.;

3: Requires personal help with washing, brushing teeth, combing hair or using toilet.

4: Fully dependent (bed-bound)

**Score: _____/4**

**Total Score: _____/32**

**eFigure 1: Flowchart illustrating patients’ selection**


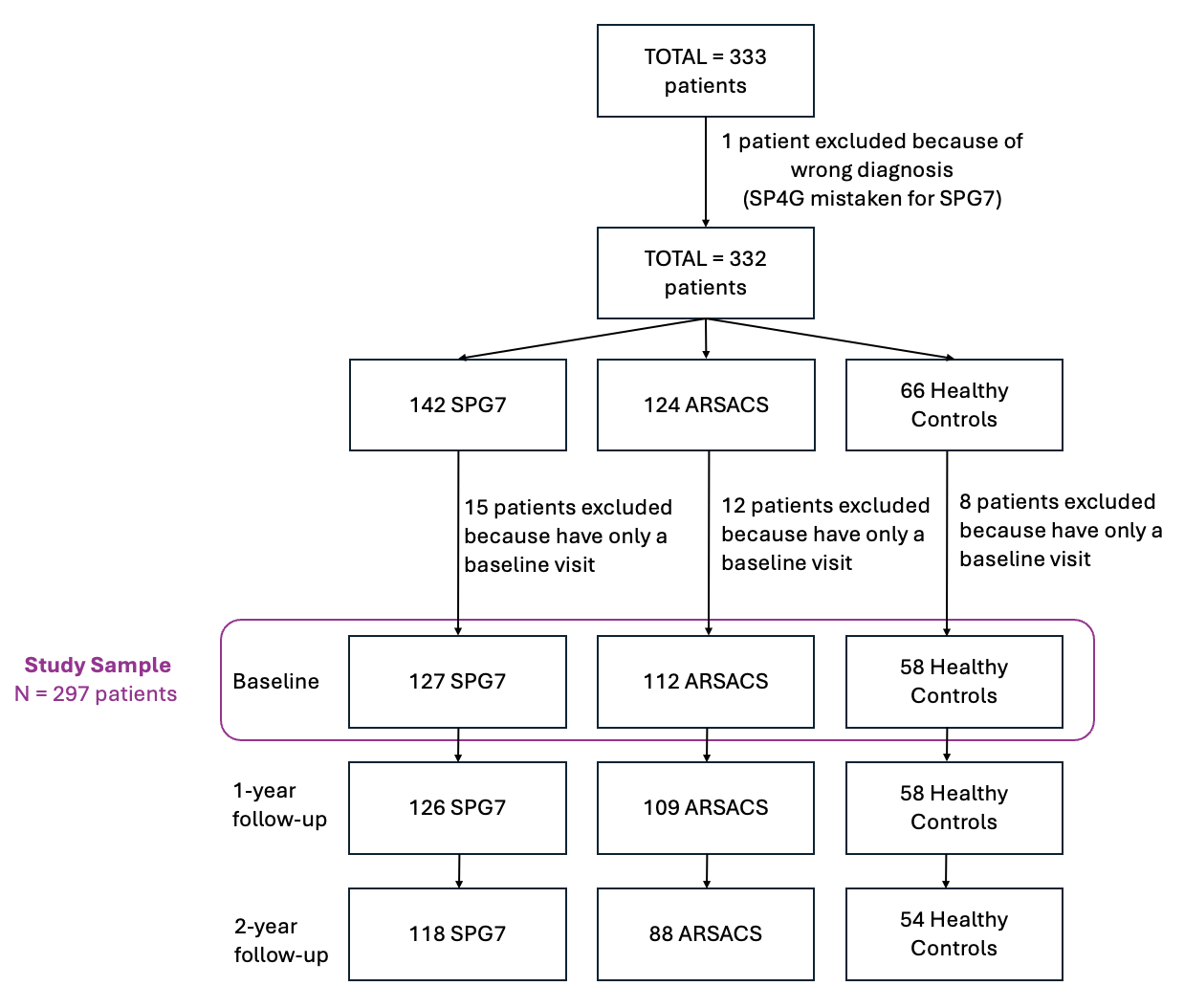


**eFigure 2: Effect size of items combinations during the stepwise procedure on 1,000 data splits.** At each iteration, the dataset is split: 60% of the data (“Training data set”) are used to select a combination of items maximizing effect size in a stepwise procedure. The effect size of this optimized combination is then assessed on the remaining 40% of the data (“Test data set”). A: We reported the effect size reached by the best combination reached at each iteration on its training and test data. B: the effect sizes of the same combinations assessed separately on the two genotypes. C: the effect sizes of the same combinations assessed separately on patients FARS-stage. The boxplot corresponding to patients with Mild stage is not shown, due to insufficient data (N=25).


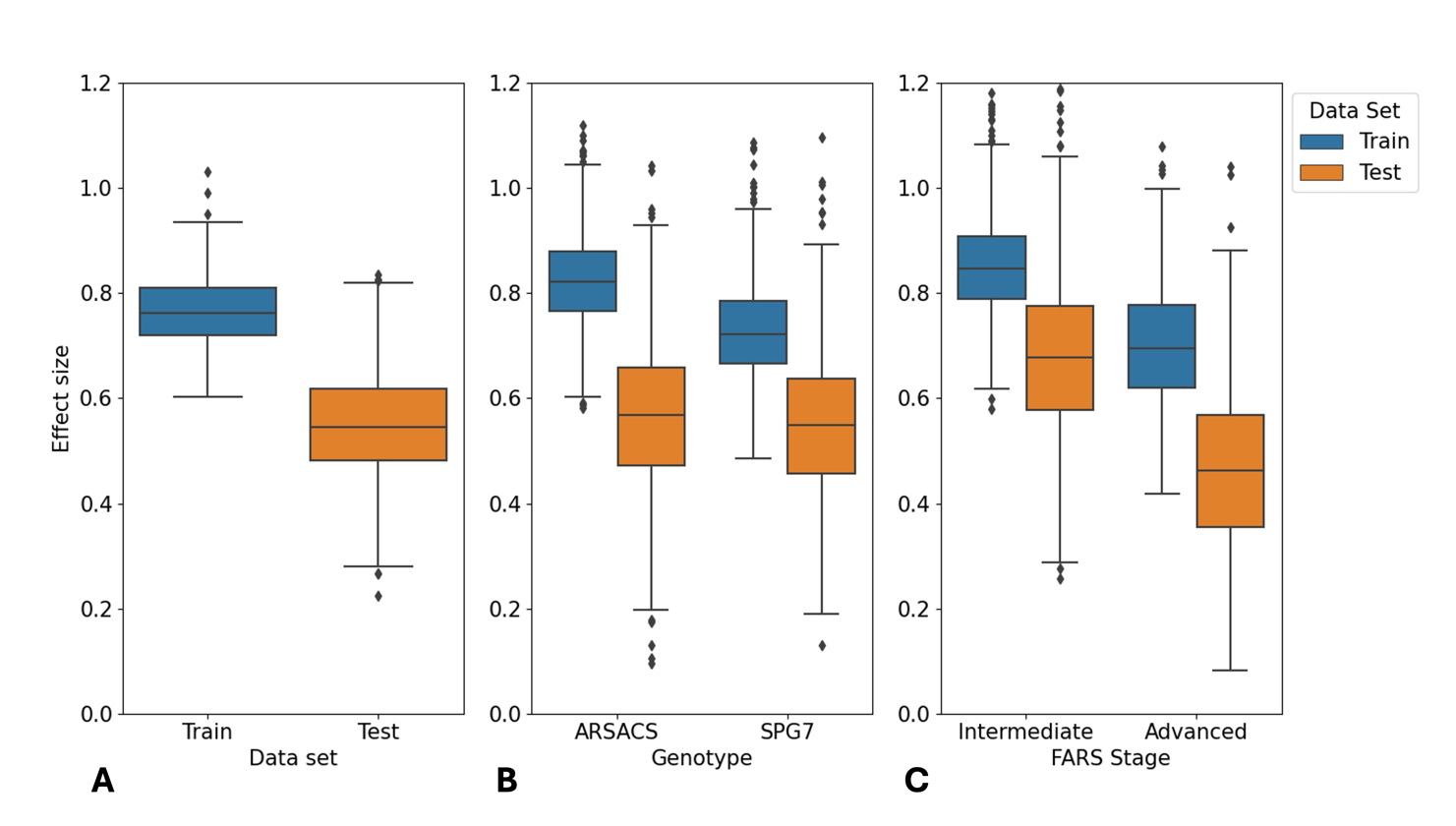


**eFigure 3: Number of times a combination of items was selected as an optimized combination (maximizing effect size) during the stepwise procedure.**

At each iteration, 60% of the data is drawn. The “optimized combination” of items, i.e., the combination maximizing effect size, is then computed in a stepwise procedure. We report the number of times each combination of items was selected as optimized over N=1,000 iterations. Over the 1,000 iterations, 161 different optimized combinations were obtained (out of 2^30^ = 1,073,741,824 possible combinations of 30 items). Consequently, only the number of occurrences of the 10 most frequent combinations are shown.


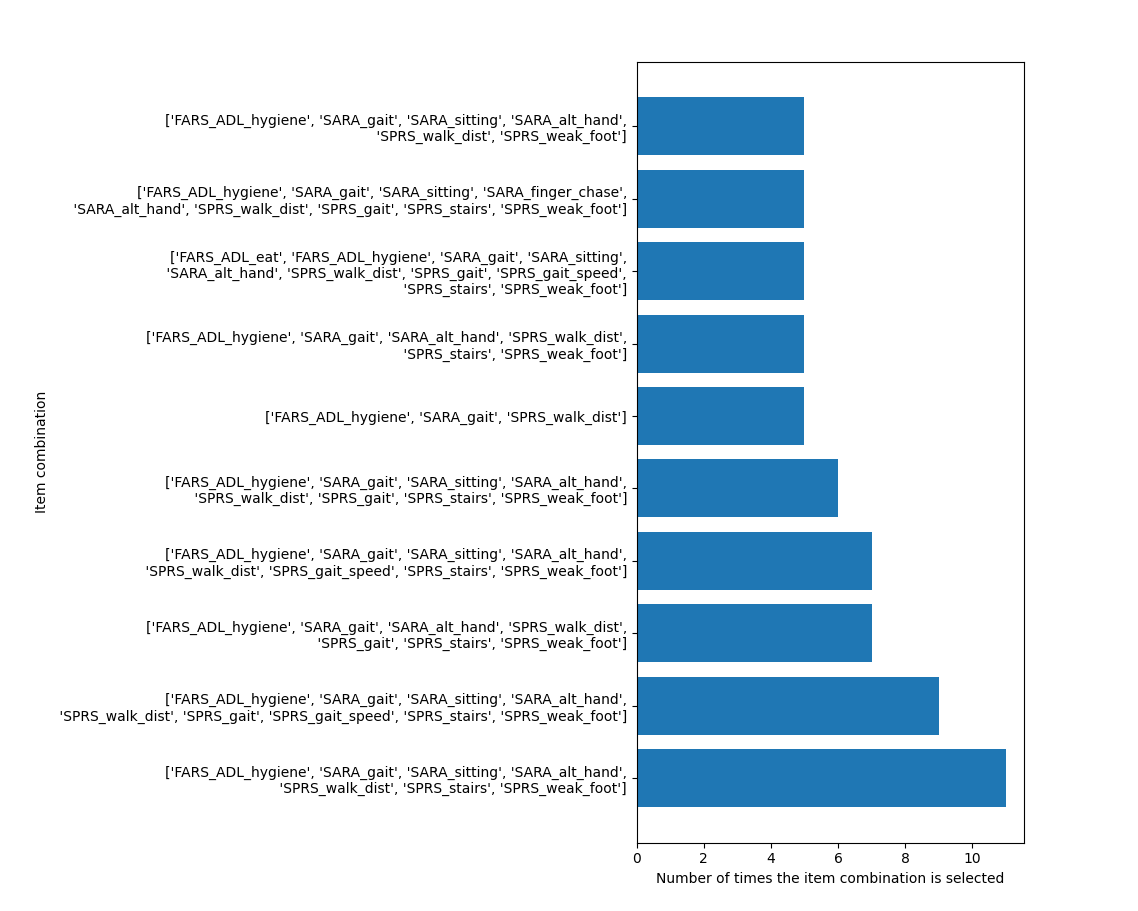


**eFigure 4: Number of times each item is selected in an optimized combination during the stepwise procedure on 1,000 data splits. At each iteration, 60% of the data is drawn. The “optimized combination” of items, i.e., the combination maximizing effect size, is then computed in a stepwise procedure. We report the number of times each item was present in the optimized combinations resulting from N=1,000 iterations.**


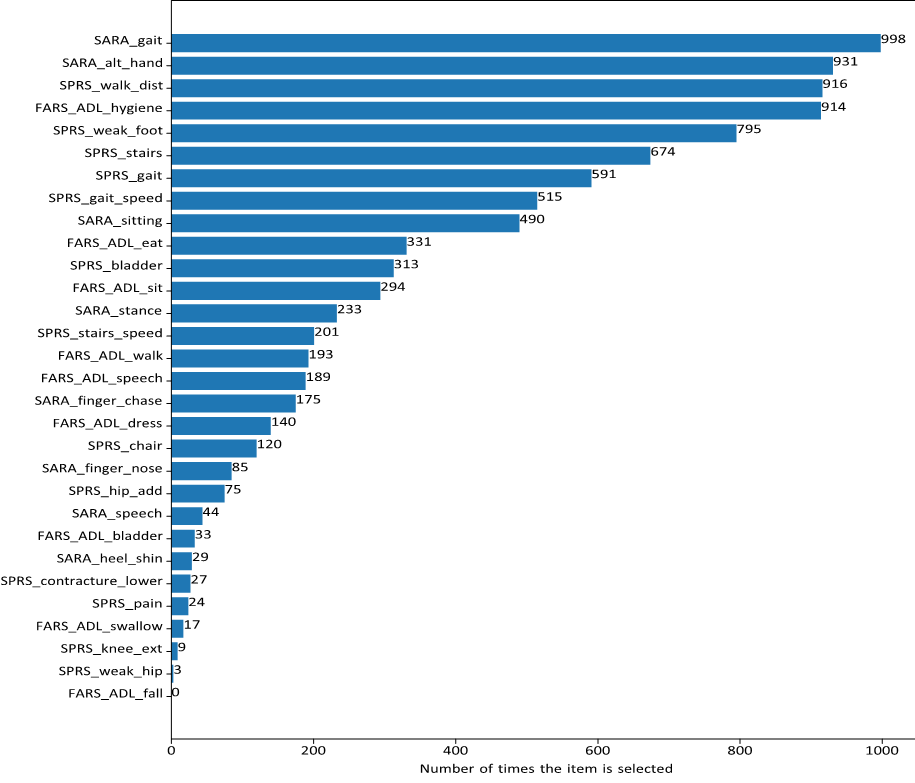


**eFigure 5: Correlation at baseline between the seven items of the SPAXCOM**


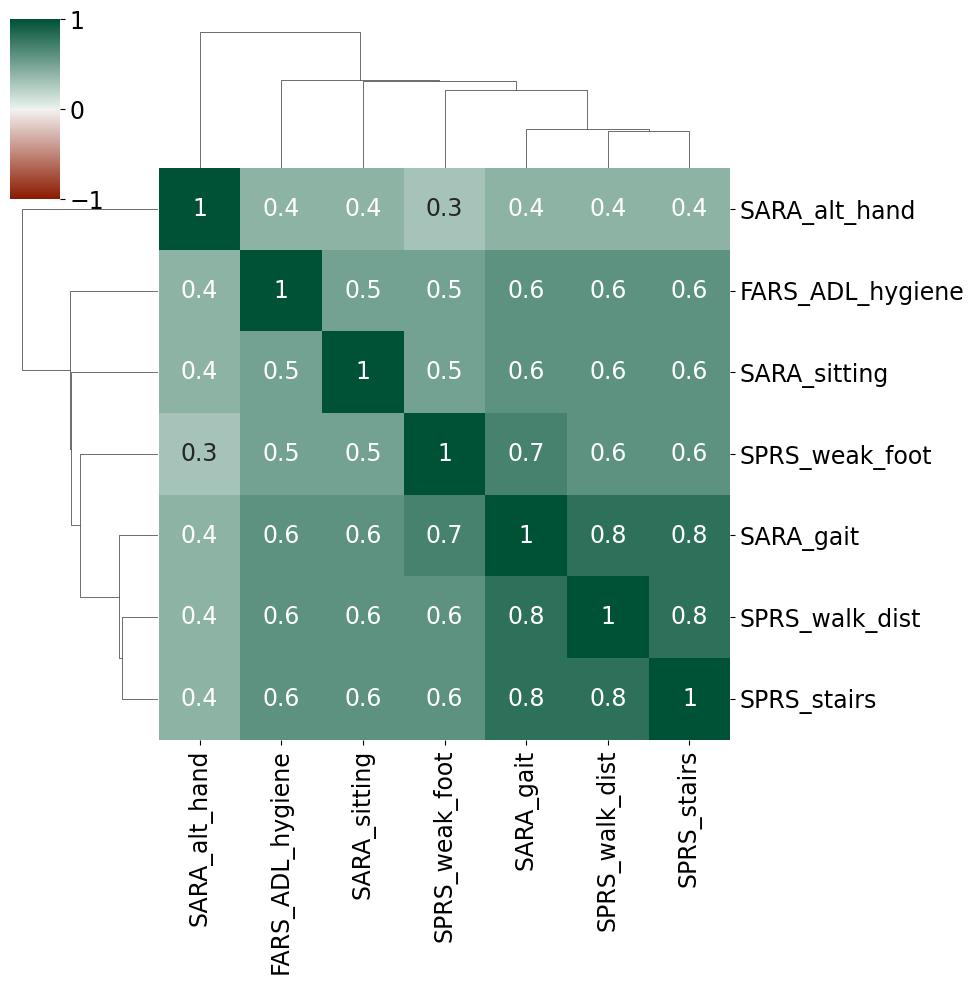


**eTable 1: Description and labels of SARA, SPRS and FARS-ADL items.**

| Scale | Item description | Item label | Max score |
| --- | --- | --- | --- |
| SARA | 1 – Gait | SARA_gait | 8 |
|  | 2 – Stance | SARA_stance | 6 |
|  | 3 – Sitting | SARA_sitting | 4 |
|  | 4 – Speech disturbance | SARA_speech | 6 |
|  | 5 – Finger chase, mean of right and left assessments | SARA_finger_chase | 4 |
|  | 6 – Finger-nose test, mean of right and left assessments | SARA_finger_nose | 4 |
|  | 7 – Fast alternating hand movements, mean of right and left assessments | SARA_alt_hand | 4 |
|  | 8 – Heel-shin slide, mean of right and left assessments | SARA_heel_shin | 4 |
| SPRS | 1 – Walking distance without pause | SPRS_walk_dist | 4 |
|  | 2 – Gait quality | SPRS_gait | 4 |
|  | 3 – Maximum gait speed | SPRS_gait_speed | 4 |
|  | 4 – Climbing stairs | SPRS_stairs | 4 |
|  | 5 – Speed of stair climbing | SPRS_stairs_speed | 4 |
|  | 6 – Arising from chair | SPRS_chair | 4 |
|  | 7 – Spasticity - hip adductor muscles | SPRS_hip_add | 4 |
|  | 8 – Spasticity - knee extensor muscles | SPRS_knee_ext | 4 |
|  | 9 – Weakness - hip abduction | SPRS_weak_hip | 4 |
|  | 10 – Weakness - foot dorsiflexion | SPRS_weak_foot | 4 |
|  | 11 – Contractures of lower limbs | SPRS_contracture_lower | 4 |
|  | 12 – Pain due to spastic paraplegia related symptoms | SPRS_pain | 4 |
|  | 13 – Bladder and bowel function | SPRS_bladder | 4 |
| FARS_ADL | 1 – Speech quality | FARS_ADL_speech | 4 |
|  | 2 – Swallowing | FARS_ADL_swallow | 4 |
|  | 3 – Cutting food and handling utensils | FARS_ADL_eat | 4 |
|  | 4 – Dressing | FARS_ADL_dress | 4 |
|  | 5 – Personal hygiene | FARS_ADL_hygiene | 4 |
|  | 6 – Falling | FARS_ADL_fall | 4 |
|  | 7 – Walking | FARS_ADL_walk | 4 |
|  | 8 – Quality of sitting position | FARS_ADL_sit | 4 |
|  | 9 – Bladder function | FARS_ADL_bladder | 4 |

**eTable 2: Effect sizes for the SPAXCOM, SARA, SPRS and FARS-ADL total scores, and their 95% bootstrapped confidence intervals.**

The row corresponding to patients with Mild FARS stage is not shown, due to insufficient data (N=25).

| **Score** | **Group** | **Effect size (CI)** |
| --- | --- | --- |
| SPAXCOM | Healthy controls | -0.12 (-0.29, 0.19) |
|  | ARSACS+SPG7 | 0.71 (0.58, 0.85) |
|  | ARSACS | 0.75 (0.58, 0.93) |
|  | SPG7 | 0.69 (0.51, 0.95) |
|  | Advanced FARS stage | 0.60 (0.43, 0.85) |
|  | Intermediate FARS stage | 0.86 (0.70, 1.05) |
| SARA | Healthy controls | -0.17 (-0.39, 0.1) |
|  | ARSACS+SPG7 | 0.43 (0.31, 0.59) |
|  | ARSACS | 0.32 (0.13, 0.54) |
|  | SPG7 | 0.59 (0.45, 0.76) |
|  | Advanced FARS stage | 0.31 (0.13, 0.54) |
|  | Intermediate FARS stage | 0.58 (0.42, 0.75) |
| SPRS | Healthy controls | 0.15 (-0.15, 0.45) |
|  | ARSACS+SPG7 | 0.42 (0.30, 0.56) |
|  | ARSACS | 0.42 (0.24, 0.61) |
|  | SPG7 | 0.42 (0.25, 0.65) |
|  | Advanced FARS stage | 0.46 (0.26, 0.69) |
|  | Intermediate FARS stage | 0.40 (0.21, 0.64) |
| FARS-ADL | Healthy controls | 0.03 (-0.22, 0.28) |
|  | ARSACS+SPG7 | 0.27 (0.14, 0.43) |
|  | ARSACS | 0.31 (0.13, 0.53) |
|  | SPG7 | 0.24 (0.05, 0.46) |
|  | Advanced FARS stage | 0.24 (0.05, 0.51) |
|  | Intermediate FARS stage | 0.31 (0.11, 0.53) |

**eTable 3: Mean annual SPAXCOM variation [95% CI] by PGI-C.**

PGI-C, Patient Global Impression-Global Change Scale. The seven levels of the PGI-C were categorized as follows: Improved (0 = Very much improved, 1 = Much improved, 2 = Minimally improved), Stable (3 = No change), Worsened (4 = Minimally worse, 5 = Much worse, 6 = Very much worse).

| **Group** | **PGI-C** | **Mean SPAXCOM variation**  **[95% CI]** |
| --- | --- | --- |
| **All patients** | Improved (N=37) | 0.44 (-0.14, 1.01) |
|  | Stable (N=108) | 0.61 (0.19, 1.03) |
|  | Worsened (N=295) | 1.22 (0.96, 1.49) |
| **ARSACS** | Improved (N=20) | 0.60 (-0.11, 1.31) |
|  | Stable (N=67) | 0.81 (0.20, 1.41) |
|  | Worsened (N=109) | 1.31 (0.89, 1.72) |
| **SPG7** | Improved (N=17) | 0.25 (-0.71, 1.20) |
|  | Stable (N=41) | 0.29 (-0.16, 0.74) |
|  | Worsened (N=186) | 1.17 (0.83, 1.51) |
| **HC** | Stable (N=76) | -0.01 (-0.10, 0.08) |

**eTable 4: Internal consistency and proportion of explained variance for the 7 items of the final SPAXCOM.**

Alpha without the item: Internal consistency is assessed by Cronbach’s alpha on baseline data (N=239), for the total set and for the set minus the item. Communality gives the proportion of explained variance by the unidimensional IRT model.

| **Item** | **Alpha without the item** | **Communality** |
| --- | --- | --- |
| **Total** | 0.878 (0.844, 0.906) | / |
| **SARA_gait** | 0.863 (0.825, 0.896) | 0.949 |
| **SARA_alt_hand** | 0.883 (0.850, 0.911) | 0.215 |
| **FARS_ADL_hygiene** | 0.861 (0.822, 0.894) | 0.570 |
| **SPRS_walk_dist** | 0.843 (0.798, 0.880) | 0.813 |
| **SPRS_weak_foot** | 0.861 (0.822, 0.894) | 0.506 |
| **SARA_sitting** | 0.866 (0.828, 0.897) | 0.596 |
| **SPRS_stairs** | 0.840 (0.795, 0.878) | 0.912 |
